# Characterization of the development of *Haemonchus contortus* ZJ strain from gerbils

**DOI:** 10.1186/s13071-017-2465-1

**Published:** 2017-10-23

**Authors:** Yi Yang, Xiaolu Guo, Hongli Zhang, Yan Huang, Xueqiu Chen, Aifang Du

**Affiliations:** 1 Institute of Preventive Veterinary Medicine, Zhejiang Provincial Key Laboratory of Preventive Veterinary Medicine, College of Animal Sciences, Zhejiang University, Hangzhou, 310058 China; 2 Zhejiang Center for Animal Disease Control and Prevention, Hangzhou, 310000 China

**Keywords:** *Haemonchus contortus* ZJ strain, Gerbil, Development, Rod-like crystalline inclusions

## Abstract

**Background:**

*Haemonchus contortus* is a serious parasitic nematode in domestic ruminants around the world, including China. *Haemonchus contortus* has developed extensive resistance to commercial anthelmintics, which has produced a demand for new control methods, such as more effective drugs. Gerbils infected with *H. contortus* have previously been used as a model for anthelmintics selections, and the growth of *H. contortus* had been briefly examined. To enhance the model, this study provides an additional description of the development of *H. contortus* ZJ strain in gerbils.

**Results:**

Gerbils were infected with *H. contortus* ZJ strain at a dose of 2000 exsheathed infective larvae (xL3s) and sacrificed at 4, 7 and 18 days post-infection (dpi). Only fourth-stage larvae were found in the stomachs. About 2% of the inoculums were obtained at each of the three sampling time points. Larvae grew more slowly in gerbils than in sheep, but presented almost the same morphology. Rod-like crystalline inclusions were present in the intestinal cells of larvae, indicating that the metabolic rate of larvae was probably greatly reduced. Histological examination of stomach sections showed that larvae are located in the lumens or at the mucosal surfaces, with few inflammatory changes evident.

**Conclusions:**

The development and features of *H. contortus* ZJ strain in gerbils were described. Our results provide supplementary information of *H. contortus* growth in gerbils, especially the presence of rod-like crystalline inclusions, and may contribute to improve the anthelmintic selection system.

## Background

The blood-feeding gastric nematode *Haemonchus contortus* is a serious pathogen in ruminants all around the world, including China. Drug resistance and lack of effective vaccines bring difficulties to the control of the disease. Previous studies have screened potential anthelmintics utilizing a *H. contortus*-infected gerbil model.

Conder et al. [1] first described the model for studying anthelmintics against *H. contortus*, and examined the growth and development of *H. contortus* in gerbils [2]; this study found that artificially exsheathed larvae were able to grow and establish successfully in gerbil’s stomach. However as compared to those in sheep, the larvae in gerbils exhibited slower and incomplete growth. De Jesus-Gabino et al. [3] and Suires et al. [4, 5] also tested the anthelmintic effects of different plant extracts on *H. contortus* in gerbils. In addition, other laboratory animals, such as mice, have been utilized to set up laboratory screening systems for anthelmintic activity against *H. contortus* [6, 7].

The slower and incomplete growth of *H. contortus* in gerbils was also observed as described in other laboratory animals. Hutchinson & Slocombe [8] found that *H. contortus* completed its development at a slower rate than in sheep when they infected laboratory rabbits with exsheathed larvae. Even though a few female worms were pregnant, a high proportion of early fourth-stage larvae were recovered and some of them contained rod-like crystalline inclusions. Wagland et al. [9] demonstrated that *H. contortus* stayed and developed into fourth-stage in guinea pigs. Mature females, earlier stage larvae and fertile eggs were found in Chinchilla [10].

Usually, sheep were chosen as a suitable host for *H. contortus* research [11], but this procedure is expensive and time-consuming. A convenient and cost effective in vivo gerbil model is urgently needed. To improve the model, this experiment details the developmental morphology of *H. contortus* ZJ strain in gerbils and provides additional description of the growth.

## Methods

### Gerbils and parasites

Gerbils (*Meriones unguiculatus*) were purchased from Zhejiang Experimental Animal Center (certificate no. scxk (Zhe) 2014-0001). The eggs of *Haemonchus contortus* ZJ strain were collected from naturally singly-infected sheep. Infective larvae (iL3s) were cultured from eggs for 7 days at 28 °C.

### *Haemonchus contortus* exsheathment and gerbil infection

The iL3s were exsheathed to xL3s using 0.2% NaOCl based on the method described by Rothwell & Sangster [12]. Thirty-three 6-month old male gerbils were selected, with body weight of 80 g each, and divided randomly into five groups: group 1 (*n* = 3), represented the control group with no treatment; group 2 (*n* = 3), the immune-depressed; group 3 (*n* = 9), the immune-depressed and infected, necropsy at 4 dpi; group 4 (*n* = 9), the immune-depressed and infected, necropsy at 7 dpi and group 5 (*n* = 9), the immune-depressed and infected, necropsy at 18 dpi. Groups 2–5 were immune-depressed by intramuscular injection with dexamethasone sodium phosphate (1 ml:5 mg, Shenniao, Bailing Animal Medicine Co., Ltd., Ganzhou, China) at a dose of 0.5 mg/kg for two consecutive days. Groups 3–5 were orally infected with 2000 xL3s.

### Necropsy of gerbils

All gerbils were euthanized and necropsy was carried out to obtain the stomachs. Six of the nine stomachs from groups 3–5 were used for larvae collection. Stomachs were digested in peptic-HCl and washed by phosphate-buffered saline (PBS) [11]. The number of larvae was counted with an anatomical lens (Motic, Fujian, China). Larvae were fixed in 7% formol saline and lengths (*n* = 10) were measured with a confocal laser scanning microscope (Olympus, Tokyo, Japan). Stomachs from groups 1–2, and three of the nine stomachs from groups 3–5, were directly fixed in 10% neutral buffered formalin for histological study.

### Histological examination of stomach

Each of the fixed stomachs was embedded in paraffin, mounted on glass sides and stained with hematoxylin-eosin (HE). Stained tissue sections were examined with an optical microscope (Olympus, Japan).

### Statistical analyses

The number and length of larvae were shown as means ± SD. A one-way ANOVA was conducted for length comparison between different groups.

## Results

Larvae were able to colonize gerbil stomachs, however, only fourth-stage larvae were found. The amount of surviving larvae was 38.7 ± 2.2, 45.0 ± 3.9 and 41.3 ± 2.8 at 4, 7 and 18 dpi, respectively (Table 1), which was 2% of the inoculums. The body lengths of larvae at the three sampling time points were 1100 ± 169 μm, 1273 ± 122 μm and 1553 ± 133 μm, respectively (Table 1). There was a significant difference between larvae recovered at 4 and 18 dpi (ANOVA: *F*_(2,9)_ = 5.221, *P* = 0.031). This suggests larvae grew slowly across infection time.

**Table 1:** The number and length (mean ± SD) of *Haemonchus contortus* larvae recovered in gerbils at necropsy on 4, 7 and 18 days post-infection

	Days post-infection		
	4	7	18
Number	38.7 ± 2.2	45.0 ± 3.9	41.3 ± 2.8
Length (μm)	1100 ± 169^b^	1273 ± 122^ab^	1553 ± 133^a^

Note: ^a^ *vs* ^b^, *P* < 0.05

**Fig. 1:**
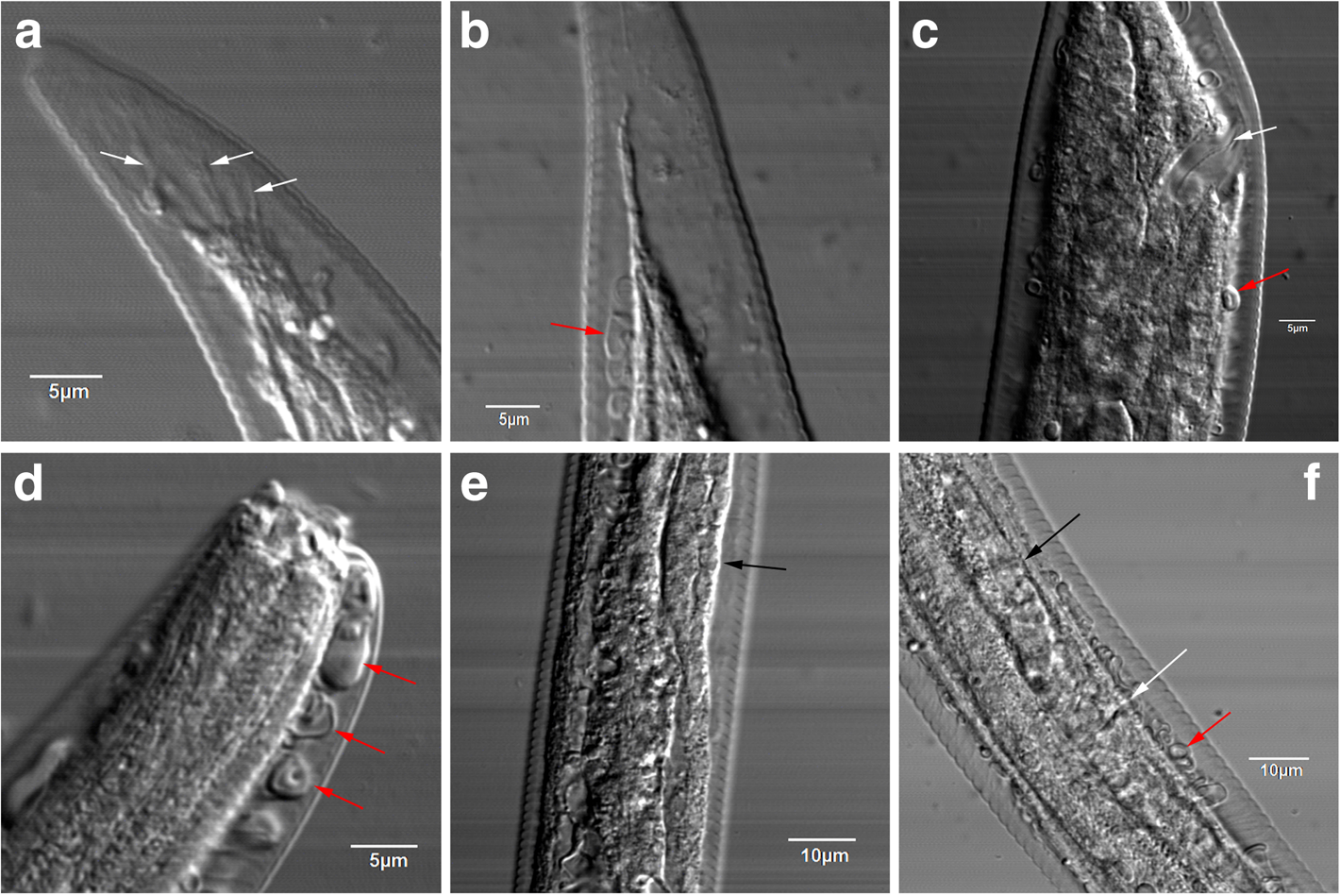
Morphological features of *Haemonchus contortus* larvae collected from gerbils. **a** The vestigial copulatory bursa (white arrows) at the posterior extremity of a male larva 4 days post-infection (dpi). **b** Posterior extremity of a female larva 4 dpi. **c** Excretory pore (white arrow) at the rear end of intestine of a female larva 4 dpi. **d** Buccal capsule at the anterior end of a female larva 4 dpi. **e** Genital primordium (black arrow) of a female larva 4 dpi. **f** Genital primordium (black arrow) and vulva ovejector primordium (white arrow) of a female larva 7 dpi. The specific tissues below the cuticle are marked with red arrows in **b**, **c**, **d** and **f**

Larvae recovered at 4 dpi displayed obvious sexual differentiation and could be identified as males and females. A swelling containing the vestigial copulatory bursa with short spicules was found at the posterior extremity of male larvae (Fig. 1a), but just a tail in the females (Fig. 1b). The well-developed excretory pore (Fig. 1c) and buccal capsule (Fig. 1d) were very prominent at 4 dpi, as was the genital primordium (Fig. 1e). Some differentiation into functional entities, such as the vulva ovejector primordium, had occurred in the genital primordium at 7 dpi (Fig. 1f). One confusing finding was the appearance of a mass of strange tissues below the cuticle. The tissues could be seen in Fig.1b-f. Most of them were helically pipe-shaped, and some were vesicle-shaped. The exact role of the specific subcutaneous substance was unknown and warrants follow-up studies.

**Fig. 2:**
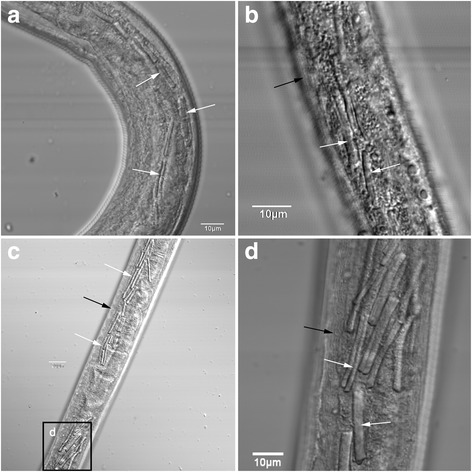
Crystalline inclusions in the intestinal cells of *Haemonchus contortus* larvae. **a** Male larva 7 days post-infection (dpi). **b** Female larva 7 dpi. **c**, **d** Female larva 18 dpi. Crystalline inclusions are indicated by white arrows, and intestinal lumen is indicated by a black arrow

The rod-like crystalline inclusions were present in *H. contortus* after 7 dpi (Fig. 2) and could be found in almost all of nematodes. The crystals were located in the intestinal cells of larvae and were fairly uniform in their rod-like shape, but varied considerably in their sizes. In some cases, two seemingly identical crystals were found parallel to each other with a narrow space. Moreover, a small number of crystals were able to split in two, giving the appearance of a Y shape. The number of crystal was found to increase with infection duration. In addition, the intestinal cells were also found to be solidly packed with crystals at 18 dpi (Fig. 2d).

**Fig. 3:**
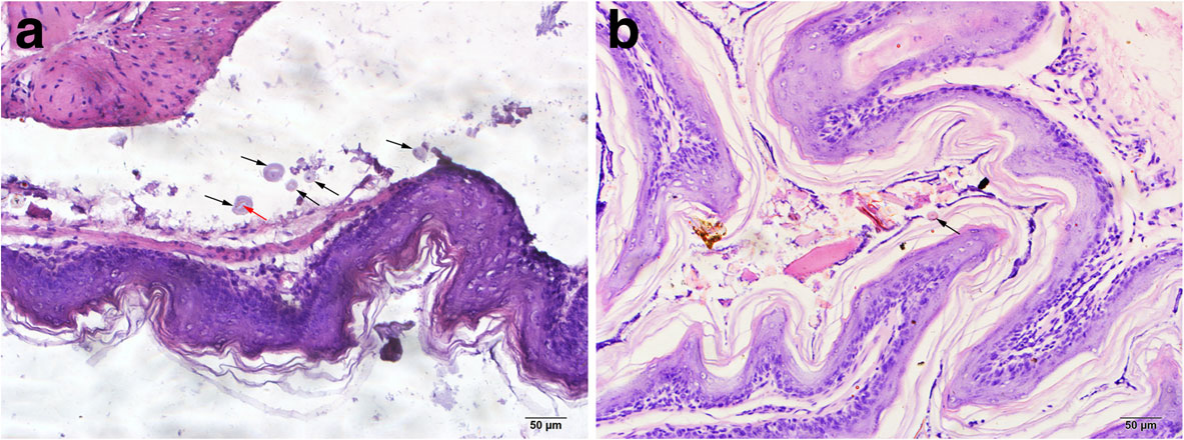
Stomach sections of gerbils. **a** Larvae located in the lumen of stomach. **b** Larvae located at the mucosal surface of stomach. Cross-sections of early fourth-stage larvae at 4 days post-infection are marked with black arrows; the intestinal lumens of a larvae is marked with a red arrow in **a**

Upon histological examination, larvae were found, in the form of cross-sections with the intestinal lumen inside, in the gerbil lumens and mucosal surfaces of stomach (Fig. 3). The presence of eosinophil infiltrates and lymphocytic aggregates, or obvious inflammatory changes, was barely detected.

## Discussion

Previous studies have made great efforts to search for a laboratory model to take the place of a ruminant model when conducting in vivo *H. contortus* infection experiments. Gerbils infected with *H. contortus* were first used as an anthelmintic screening model [1]. This model provided useful and credible information about host-parasite interactions. Rodent models, not just gerbil, including mouse, hamster, chinchilla and rabbit, could improve discovery efficiency, which means more work could be done prior to being completed in ruminants. Small laboratory animals offer convenience in feeding and handling, as well as lowering the research cost.

The amount of *H. contortus* recovered from gerbils can reach up to more than 30% of the inoculums [2]. However the larvae that survived in our experiment accounted for only a small percentage (2%) of the inoculums. The factors causing this difference might be various; the inoculation dose of larvae is probably the most important one. Conder et al. found that inocula of 500–2000 xL3s resulted in relatively constant worm recoveries, which kept 5–30% of the inoculums, and a dose of 1000 larvae was chosen for further work [2]. Squires et al. [4, 5] gave 600 xL3s to gerbils and about 10% larvae recovered. But unlike the above two experiments, De Jesus-Gabino et al. [3] administered 4000 sheathed larvae to each gerbil, and the recovery rate was just 1.6–2.0%. The relationship between infection dose and recovery rate seems to be unpredictable and occasional. Other factors, such as larval exsheathment method, sex and age of gerbils, the individual difference of host and environmental factors, were all other possible factors. Further study is required to enhance the recovery rate of *H. contortus*.

According to the life-cycle described by Veglia [13] (cf. Georgi & Georgi [14]), once ingested by the appropriate host, a ruminant for *H. contortus*, the L3 exsheath and grow to early L4 at ~4 dpi, and then L4 at ~7 dpi. Specialized development of the buccal capsule for blood-feeding and sexual differentiation is available at the fourth stage, following by gametogenesis in the adult stage. Female adults lay eggs at ~18 dpi, following copulation with males. The body length of larvae from L3 to adult could be ~1 mm to ~15–18 mm (male) or to ~25–30 mm (female). In this experiment, the basic tissue differentiation and body length of larvae in gerbils was similar to that of normal larvae in sheep at 4 dpi. However larvae recovered from gerbils at 7 and 18 dpi were significantly shorter than those in sheep at the same time point [2]. This was probably induced by host differences, since the gastric conditions, including pH, gas composition and content, of gerbil and sheep are very different [15–18]. Even the growth extent was much lower than that found by Conder et al. [2], which also described a gerbil model. Larvae grew to the length of 1100 ± 169 μm, 1273 ± 122 μm and 1553 ± 133 μm at 4, 7 and 18 dpi, respectively, in our study, but grew to 1320 ± 333 μm, 2055 ± 290 μm (male) and 5415 ± 3589 μm (male) at 4, 7 and 21 dpi, respectively, in the study of Conder et al. [2]. This could be explained by a different parasite strain and housing environment.

*Haemonchus contortus* can undergo arrested development at the fourth stage in sheep abomasum when threatened by host or environment [19, 20]. Arrest also refers to diapause in *H. contortus*. Diapause is defined by two unique characteristics [11, 15]. First, it is induced by environmental cues, often seasonal changes, and the arrest is temporarily irreversible. Secondly, the body length of diapausing larvae is short and uniform. Resumption of development will not proceed unless diapause development has been completed and environmental conditions are favorable. In *H. contortus*, diapause ensues when infective larvae (iL3s) undergo development for 3 or 4 days to reach the early fourth-stage after host infection [21]. Larvae recovered from gerbils in our experiment were not uniform, although they were short. The most important point was that larvae became longer with increased infection length. Thus, they just grew at a much slower speed in an inappropriate host, rather than enter into diapause.

Another symbolic feature of diapausing *H. contortus* is the presence of rod-like crystalline inclusions in the intestinal cells [21]. While these inclusions can still be found in the adult stage when the diapausing larvae have resumed development, they were not detected in normally developing larvae. In this study, the rod-like crystalline inclusions appeared in the intestinal cells at 7 dpi and were present from this time on. This is a new discovery which was absent in the study of Conder et al. [1, 2]. Earlier work has reported the presence of protein and sulphur in the crystals [15], while our recent DAPI staining results revealed positive nucleic acid staining [11]. Although the significance of these compounds remain unknown, it is possible that they were built up metabolic waste products generated during the process of development [21, 22]. Colglazier et al. [23] supported this theory, in which diapausing *H. contortus* conferred great resistance to most anthelmintics including thiabendazole. As such, it is reasonable that the number of crystals increased with infection days, as the metabolic rate and energy requirements were probably greatly reduced in slowly developing *H. contortus*. Rod-like crystalline inclusions were also present in *H. contortus* recovered from rabbits, but without any explanation to this phenomenon [8].

Since larvae were mostly located in the lumens or on the stomach mucosal surfaces, eosinophils or other inflammatory responses were not obvious in our results. We performed routine peripheral blood examination after gerbil infection, and found few changes in the numbers of red blood cells, eosinophils or lymphocytes (data not shown). This indicated that, unlike sheep, gerbils infected with *H. contortus* failed to establish a significant systemic immune response, or even a local reaction. It is possible that *H. contortus* were being regarded as food items and subsequently digested in non-routine hosts. Only the most adaptive larvae survived, but they failed to develop to adulthood. In addition, it is possible that the small number (about 2% of the inoculums in our study) of larvae was insufficient to elicit effective immune responses. Gerbils were immunosuppressed by dexamethasone, and therefore this may also affect their immune responses. A higher larval dose would be required to stimulate a local reaction [2].

## Conclusions

The present study has successfully obtained fourth-stage *H. contortus* larvae in a gerbil infection model. Morphological characterization of the larvae revealed shorter larval length but almost the same appearance, which suggested that slower development, rather than arrested development, has taken place in gerbils, compared to in sheep. The presence of rod-like crystalline inclusions in nematode intestinal cells was found, indicating that slowly developing *H. contortus* had a reduced metabolic rate. Our results provide an additional description of the development of *H. contortus* ZJ strain from gerbils and may contribute to enhance the anthelmintic screening model.

## Abbreviations

ANOVA: Analysis of variance
dpi: Days post-infection
HCl: Hydrochloric acid
HE: Hematoxylin-eosin
iL3s: Infective larvae
L3: Third-stage larvae
L4: Fourth-stage larvae
SD: Standard deviation
xL3s: Exsheathed infective larvae
ZJ: Zhejiang
